# Hidradenoma papilliferum of the external auditory canal. Case report

**DOI:** 10.1016/j.amsu.2019.11.002

**Published:** 2019-11-15

**Authors:** Rabii Laababsi, Zineb Elkrimi, Anass Bouzbouz, Adil Lekhbal, Sami Rouadi, Reda Abada, Mohammed Roubal, Mohammed Mahtar

**Affiliations:** ENT Department, Face and Neck Surgery, Hospital August, 20'1953, University Hospital Centre IBN ROCHD, Casablanca, Morocco

**Keywords:** Hidradenoma papilliferum, Externalear, Case report

## Abstract

**Introduction:**

Hidradenoma papilliferum, also known as papillary hidradenoma, is a rare tumor of the sudoral glands, that occurs almost exclusively in women, on the vulvar and perineal region. Non-genital papillary hydradenoma is an even more rare occurrence, though some cases have been described in literature.

This is a report of a 56 years old patient who presented with unilateral hearing loss secondary to a mass of the external auditory canal (EAC). A biopsy allowed us to make the diagnosis of a papillary hidradenoma of the EAC. The treatment was based on surgery; the tumor was removed while respecting the adjacent structures. Follow-up of the patient shows no actual recurrence.

**Conclusion:**

Ectopic Hidradenoma papilliferum located in the EAC is a rare occurrence, and should be considered as a possible diagnosis when investigating a mass of the EAC.

## Highlights

• Hidradenoma papilliferum is typically a benign genital tumour, there have been reported cases of an ectopic localization in the head and neck region.
• The principal differential diagnosis of a mass of the EAC should be a malignant tumor, particularly a squamous cell carcinoma.
• Surgery remains the first-line treatment of benigntumours of the EAC. The extent of the the excision will depend on the size of the tumour and its histological type.

## 1 Introduction

Hidradenoma papilliferum is a benign, slow-growing tumor originating from sudoral glands, with apocrine differenciation [1]. It mainly affects women, as itis found mostly in the vulvar and perineal skin, although there have been reports of rare cases where the tumor occurred in another localization [2,3].The most common localization for non-genital hidradenoma papilliferum is the head and neck [1]. In this article, we describe the case of an ectopic hidradenoma, located in the external auditory canal (EAC). This case has been reported in line with the SCARE criteria [4].

In the case of the external auditory canal (EAC), the tumor develops from the ceruminous glands, which are modified apocrine sweat glands [5]. The main symptom of hidradenomas as in this case is conductive hearing loss. The diagnosis is initially based on clinical examination, though it is ultimately confirmed by histopathology. Treatment is based on a surgical procedure to remove the tumor while preserving the surrounding structures.

## 2 Case report

A 56 years old north african male patient, with no prior medical condition, presented in our department with a 5-month history of hearing loss. There was no notion of facial palsy or otorrhea.

**Fig. 1:**
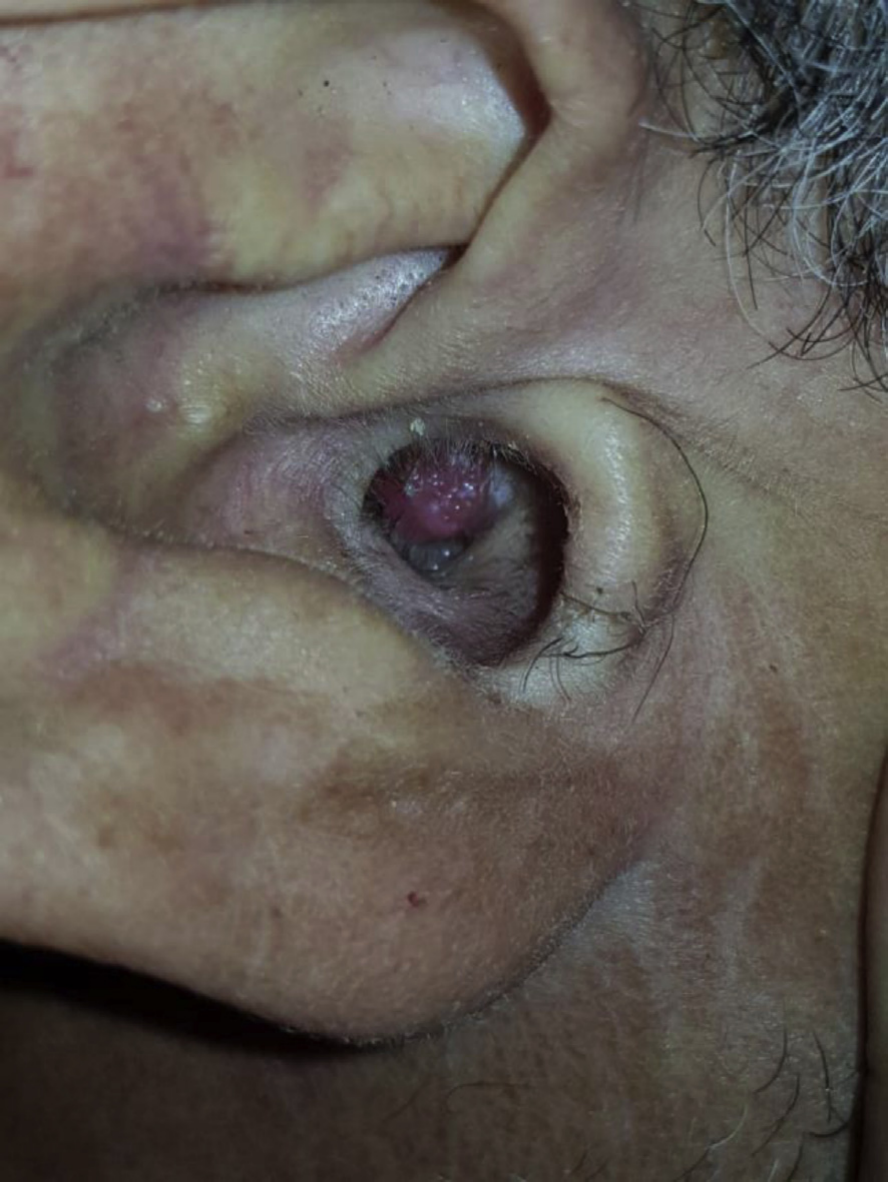
Ectopic hidradenoma papilliferum located in the right EAC, appearing as a polypoid burgeoning mass.

**Fig. 2:**
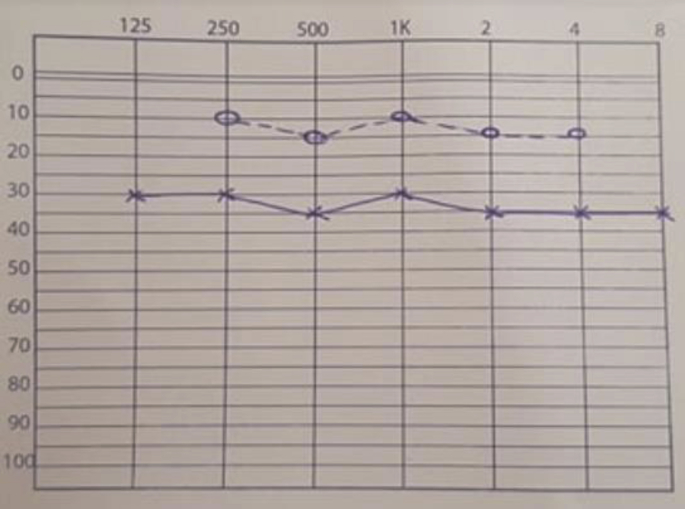
**Image of the PTA of the right ear, showing a conductive hearing loss with a hearing threshold of 35 dB**.

Clinical examination found a red burgeoning mass in the right EAC, measuring approximatively 1,5 cm in diameter (Fig. 1). Pure Tone Audiometry (PTA) found a conductive hearing loss of the right ear, with a hearing threshold of 35 dB, with a Rinne of 20 dB (Fig.2). The left ear had normal hearing. We first thought of a malignant tumor, mainly a squamous cell carcinoma, and so we performed a CT scan to evaluate local extension, and a biopsy to establish the histopathological diagnosis.

Computed tomography showed images pertaining to a tumor of tissular density (Fig. 2), limited to the external auditory canal, reaching the tympanic membrane, without bone erosion. The middle ear was intact.

**Fig. 3:**
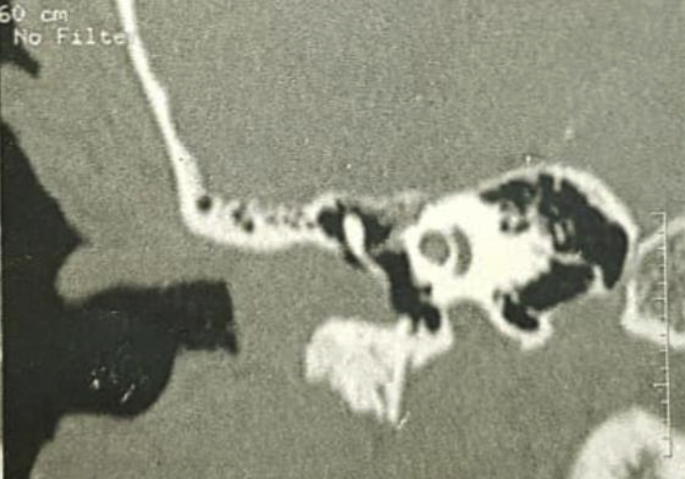
**CT image showing a tissular tumor of the EAC. The middle ear and the temporal bone are intact**.

**Fig. 4:**
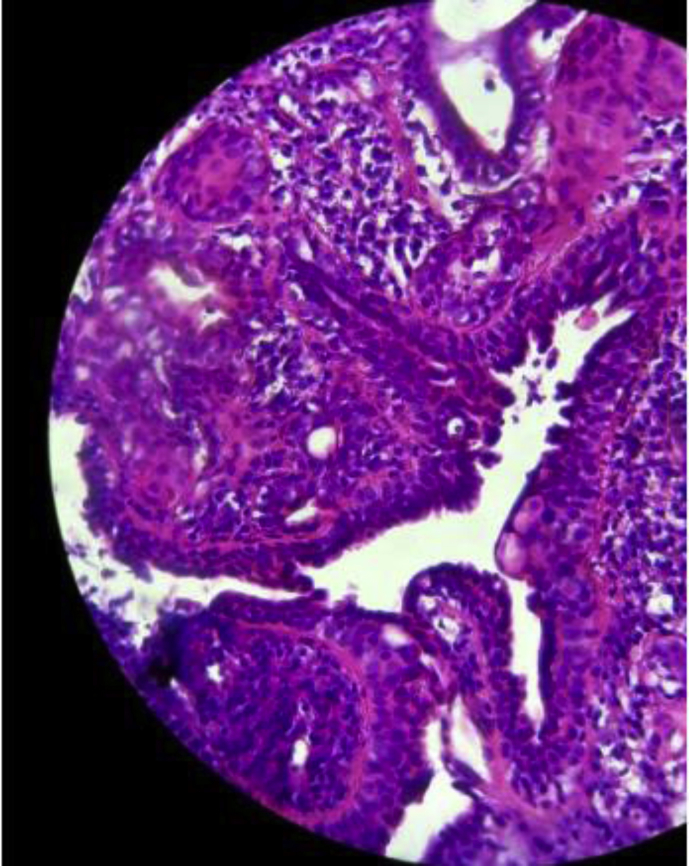
Hidradenoma papilliferum ; a double layer of cells is seen : basal cuboidal cells and luminal columnal cells

A biopsy realised under local anesthesia allowed us to diagnose a papillary hidradenoma of the EAC (Fig. 3), showing the presence of many papillary structures, covered with a double layer of internal cylindric cells and externalcuboïd cells. (See Fig. 4)

The patient received surgical treatment a month after the first consultation.Surgery was performed under general anesthesia, using a combined retroauricular and endaural approach.The intervention consisted of the complete resection of the mass, while preserving the tympanic membrane and the auricle.

A 3-month follow up found the patient in good health, with no clinical sign of a relapse.

## 3 Discussion

Ceruminous glands are modified sudoral glands, localized deep in the skin of the EAC [[5], [6], [7]]. Tumors of these glands, commonly grouped under the name « ceruminoma », are rare, and comprise 5% of EAC tumors [[8], [9], [10]]. These tumors can usually be divided into benign (such as: adenoma; pleomorphicadenoma; syringocystadenoma …) and malignant tumors (for example: adenoidcystic and mucoepidermoid carcinoma; adenocarcinoma) [5,8,11].

Hidradenoma papilliferum is a benign, cystic tumor, which characteristically occurs in the anogenital region, mostly in white women [2]. However, a few cases of ectopic hidradenoma papilliferum have been described, with the head and neck region being the most frequent localization, and half of these patients were men [1,2], like our patient.

Ectopic hidradenoma papilliferum localized in the EAC usually presents as a unilateral conductive hearing loss, sometimes associated with otalgia, otorrhea or facial palsy [5,8,10,12].These symptoms are common to all masses of the EAC, making the clinical diagnosis of hidradenoma difficult. In the case we report, our patient presented with conductive hearing loss alone. A polypoid mass of the EAC was found upon clinical examination [7]. The tympanic membrane is partially or totally covered by the tumor, without be in gengulfed by it.

Computed tomography is especially useful when a malignant tumor is suspected, to assess the type of tumor and its spreading to the local structures (temporal bone, middle ear), as well as to determine the anatomical relationships of the tumor while preparing for surgery [8]. In the case of hidradenoma papilliferum, computed tomography will find a tissular lesion of the EAC without loco-regional invasion.

Confirmation of the diagnosis relies solely on histology [1], though the diagnosis itself may be problemtic, due to the varied histological aspects and the variable names used to describe the different types of ceruminomas [5].Hidradenoma papilliferum is recognized by the presence of a cystic eosinophilic space, associated to papillary and adenomatous structures, lined with a double layer of epithelial cells [1,11]. The basal layer contains cuboidal cells, while the luminal layer is made of large columnar cells [2].As this type of hidradenoma is benign, neoplastic cells shouldn't be found.

It is recommended that the type of surgery should be adapted to the histological type of the tumor [5,10]. The surgical technique used in our patient consisted of removing the entire lesion without damaging the tympanic membrane and the auricle. More aggressive types of surgery, as well as complementary radiotherapy, maybe proposed in the case of a malignant tumor, which, contrary to a benign tumor, has a high potential for local extension and a high incidence of reccurence, necessitating a regular follow-up [5,7,10,13].

## 4 Conclusion

Although rare, the possibility of sweat gland tumors, particularly malignant tumors, should be considered when investigating a mass of the EAC, to enable early diagnosis and treatment. Radiological evaluation and pathological examination are vital to make the diagnosis. The treatment relies mainly on surgical excision, which should be wide to prevent a local reccurrence.

## Ethical approval

Written informed consent for publication of their clinical details and/or clinical images was obtained from the patient.

Ethical approval has been exempted by our institution.

## Sources of funding

None.

## Author contribution

Rabii LAABABSI: Corresponding author writing the paper.

Zineb ELKRIMI: writing the paper.

Boouzbouz anass: writing the paper.

Lekhbal adil: writing the paper.

Reda Abada: study concept.

Sami Rouadi: study concept.

Mohamed Roubal: correction of the paper.

Mohamed Mahtar: correction of the paper.

## Trial registry number

Researchregistry5198.

## Guarantor

Laababsi rabii.

## Provenance and peer review

Not commissioned, externally peer reviewed.

## Declaration of competing interest

The authors declare having no conflicts of interest for this article.

## Appendix A Supplementary data

scare_2018_guidelines scare_2018_guidelines
